# Double Immunochromatographic Test System for Sensitive Detection of Phycotoxins Domoic Acid and Okadaic Acid in Seawater and Seafood

**DOI:** 10.3390/mi13091506

**Published:** 2022-09-10

**Authors:** Olga D. Hendrickson, Elena A. Zvereva, Olga N. Solopova, Anatoly V. Zherdev, Peter G. Sveshnikov, Sergei A. Eremin, Boris B. Dzantiev

**Affiliations:** 1 Bach Institute of Biochemistry, Research Center of Biotechnology, Russian Academy of Sciences, Leninsky Prospect 33, 119071 Moscow, Russia; 2 Blokhin National Medical Research Center of Oncology, Ministry of Health of the Russian Federation, Kashirskoye Shosse 24, 115478 Moscow, Russia; 3 Russian Research Center for Molecular Diagnostics and Therapy, Sympheropolsky Blvrd., 8, 117638 Moscow, Russia; 4 Faculty of Chemistry, Lomonosov Moscow State University, Leninskie Gory, 119991 Moscow, Russia

**Keywords:** marine toxins, immunochromatographic analysis, seafood, seawater, food security

## Abstract

In this investigation, a double immunochromatographic analysis (ICA) of two relevant phycotoxins, domoic acid (DA) and okadaic acid (OA), was developed for the first time. The ICA was performed in the indirect competitive format using gold nanoparticles conjugated with anti-species antibodies. Under optimal conditions, the instrumental detection limits/cutoffs for simultaneous detection of DA and OA were 1.2/100 and 0.1/2.5 ng/mL, respectively. The time of the assay was 18 min. The ICA was applied to test seawater and a large panel of seafood, including mussels, tiger shrimps, octopuses, whelks, crabs, and scallops. The proposed simple sample preparation method for seafood takes only 20 min. For seawater, a dilution by buffer was implemented. The assay recoveries varied from 80.8% to 124.5%. The competitive potential of the proposed technique as a tool to control natural water and seafood samples is determined by its simplicity, rapidity, and sensitivity.

## 1. Introduction

The traditional kitchens of many countries include food products based on marine organisms, such as fish, shellfish (mussels, squids, octopuses, oysters, etc.), and crustaceans (crabs, shrimp, lobsters, etc.). Due to their high nutritional value, excellent taste properties, and delicacy, they are typical fishery and aquaculture products around the world. However, fish and seafood can be contaminated by substances hazardous to human health, some of which are phycotoxins—a group of marine toxins produced by certain types of algae, microalgae, and cyanobacteria [1,2]. Marine macroorganisms are capable of accumulating phycotoxins, being an intermediate link in the food chain between toxin-producing microorganisms and humans consuming mollusks and fish. When entering the human body, phycotoxins can cause both acute poisonings, accompanied by specific symptoms, and chronic toxicity, including carcinogenesis, teratogenicity, hepatotoxicity, etc. [3,4]. At the same time, traditional cooking methods do not destruct phycotoxins.

The basis for the classification of phycotoxins is the nature of the clinical picture of the poisoning they cause. Phycotoxins belonging to the diarrhetic shellfish poisoning (DSP) group provoke abdominal pain, diarrhea, nausea, vomiting, fever, headache, and chills; long-term effects include gastrointestinal tumors and other adverse effects [5]. The DSP phycotoxins include okadaic acid (OA)—a lipophilic polyester compound produced by marine microscopic algae dinoflagellates belonging to the genus *Dinophysis* [6]. The representative of the amnesic shellfish poisoning (ASP) group is domoic acid (DA), an amino acid that is produced by the diatoms *Nitzschia pungens*, *Pseudo-nitzschia*, and the red algae *Chondria armata* [7,8]. In addition to digestive disorders (liver inflammation, abdominal pain, vomiting, diarrhea), ASP toxins provoke serious neurological problems—confusion, disorientation, memory loss, seizures leading to coma and death, and anterograde memory deficit [9,10]. Given the particular threat to human health, the absence of phycotoxins in fisheries is an indicator of food safety. According to European Union legislation, requirements for mandatory control in seafood for the concentration of DA and OA have been introduced [11]. DA content in mollusks should not exceed 20 mg/kg. For OA equivalent (including OA, DTX1, and DTX2), the regulatory limit is set to be 0.16 mg/kg in shellfish.

It should be noted that the areas of harmful algal blooming (the so-called “red tides”) are almost worldwide, and under favorable conditions, the generation of microscopic algae can occur over vast areas [12]. To prevent economic damage and to ensure a guaranteed market and competitive production in mariculture farms, the control of the emergence and development of harmful algae in all places of cultivation and production of shellfish and fish, as well as monitoring of food products for the presence of phycotoxins, is of great importance.

In this regard, rapid simultaneous control of several relevant phycotoxins belonging to different groups of shellfish poisoning is of particular relevance. Multiplex determination increases testing productivity and reduces costs, time, reagents’ consumption, and the amount of detected sample. Methods that allow rapid monitoring of several samples for the presence of marine toxins include immunochromatographic analysis (ICA), based on the principles of lateral flow and immune recognition [13]. Compared to microplate immunoassay (ELISA) and other immunochemical methods, ICA is most adapted for the autonomous use of test systems without additional equipment, which confirms its widespread use for point-of-care testing. All processes on the immunochromatographic test strip are provided by the movement of reagents through the pores of the membranes under the action of capillary forces and do not require operator intervention. ICA is a very simple procedure requiring minimal skill and can be performed rapidly (in most cases, testing takes 10–20 min). In addition, ICA-based test systems demonstrate high sensitivity and allow for obtaining not only qualitative (“yes/no”) but also semiquantitative and quantitative results. The presence or absence of coloration of certain areas of the test strip as a result of the analysis can be assessed with the naked eye, which makes ICA a widely applied approach for primary screening.

The ICA of phycotoxins is widely described in the scientific literature, and among the determined phycotoxins, there are representatives of different classes [14,15,16,17,18]. For OA and, to a lesser extent, for DA as key marine toxins, immunochromatographic test systems have also been proposed, which in most cases are limited to detection in any one real object [19,20,21,22,23,24]. At the same time, the multiplex determination of phycotoxins is limited to only two works—the study of Ling et al. (2019) on the simultaneous detection of OA and neurotoxin tetrodotoxin (TTX), and the investigation of Zhang et al. (2019), which developed the ICA of OA and microcystin-LR (MC-LR) [25,26]. It may be worth noting the interesting study by Xing et al. (2015), who created a multiparametric analysis of five compounds, one of which belonged to phycotoxins (MC-LR) [27]. Ling et al. (2019) developed test strips using gold nanoparticles (AuNPs) conjugated with monoclonal antibodies (MAbs) against OA and TTX (direct competitive ICA) [25]. The cutoffs were 0.75 ng/mL for OA and 15 ng/mL for TTX, and the testing time was 10 min. The assay was tested to determine TTX and DA in clams and green mussels. Zhang et al. (2019) also developed a direct multiplex ICA based on commercial red- and green-colored fluorescent microspheres as a label [26]. The ICA had limits of detection (LODs) equal to 0.074 μg/kg for MC-LR and 2.42 μg/kg for OA in fish samples. The time of the assay was 20 min.

Despite the important advances made in the last few years, the availability of rapid tests for the screening of most common marine biotoxins currently regulated in most countries is still limited. The complexity of biological matrices makes this application even more challenging since complex and tedious sample pretreatment is typically required to remove interfering compounds. Thus, to date, test systems for the simultaneous determination of OA and DA as representatives of two key phycotoxin groups (DSP and ASP) that are a priority in ensuring the food safety of seafood have not been reported.

Here, we have developed the first system for the rapid and sensitive multiplex detection of OA and DA based on a competitive indirect assay with AuNPs as a marker for anti-species antibodies. The panel of real objects was much broader than in previous studies devoted to the individual and multiplex determination of OA and DA, and it included not only several representatives of mollusks and crustaceans but also seawater samples as the primary target of phycotoxins’ contamination. The matrix preparation procedure was designed so that the time from receiving real samples to obtaining results was less than 1 h.

## 2. Materials and Methods

### 2.1. Reagents and Materials

Gold(III) chloride hydrate, DA, OA, MC-LR, soybean trypsin inhibitor (STI), bovine serum albumin (BSA), methanol, sucrose, sodium azide, Triton X-100, dimethyl sulfoxide (DMSO), 3,3′,5,5′-tetramethylbenzidine dihydrochloride (TMB), N-hydroxysuccinimide (NHS), and N-(3-dimethylaminopropyl)-N’-ethyl-carbodiimide hydrochloride (EDC) were from Sigma-Aldrich (Saint Louis, MO, USA). Goat anti-mouse and donkey anti-goat immunoglobulins (GAMI and DAGI) were from Arista Biologicals (Allentown, PA, USA). Peroxidase-labeled GAMI (GAMI–HRP) were from Jackson Immuno Research Labs (West Grove, PA, USA). MAbs against OA (clone 7E1) were from Santa Cruz Biotechnology (Dallas, TX, USA). According to the manufacturer, the MAbs have strict specificity to OA and do not interact with its structural analogs, such as dinophysistoxin-1, dinophysistoxin-2, dinophysistoxin-3, and pectenotoxin-2. All other compounds were analytically pure. Polystyrene 96-well transparent microplates Costar 9018 from Corning Costar (Tewksbury, MA, USA) were used for the ELISA.

### 2.2. Synthesis of OA–Protein and DA–Protein Conjugates

Conjugates of OA and DA with BSA and STI were prepared by slightly modified protocols of Wang et al. (2017) and Finlay et al. (2006), respectively [28,29]. To obtain DA–protein conjugates, EDC (20 µL, 25 mg/mL) and NHS (30 µL, 30 mg/mL) in DMSO were added to DA (1 mg in 50 µL of DMSO). The mixture was shaken for 1.5 h at room temperature (RT). Then, STI or BSA (5 mg in 0.25 mL of 85 mM borate buffer, pH 9.0) was added and shaken for 1.5 h again.

To synthesize OA–protein conjugates, three solutions in DMSO, namely EDC (100 μL, 5 mg/mL), NHS (100 μL, 9 mg/mL), and OA (200 μL, 5 mg/mL) were mixed and vortexed for 30 min at RT. Then, STI or BSA (400 μL, 5 mg/mL or 2.5 mg/mL, respectively, in 50 mM carbonate buffer, pH 9.5) was added dropwise and shaken for 2 h at RT. Then, the final solutions were dialyzed for 16 h at 4 °C against 50 mM K-phosphate buffer, pH 7.4, with 0.1 M sodium chloride (PBS).

### 2.3. Production of MAbs

Anti-DA MAbs were produced using female BALB/c mice (1.5–2 months) by hybridoma technology with DA–STI as an antigen [30]. Studies in animals were performed following the EU Directive 2010/63/EU and authorized by the Ethics Committee of the Research Center of Biotechnology (protocol N22-D dated 12 February 2020).

### 2.4. ELISAs of OA and DA

As coating antigens, DA–BSA or OA–BSA (both 1 μg/mL, 100 μL in PBS) was adsorbed in the microplate wells overnight at 4 °C. Every step of the ELISA was followed by fourfold washing of the wells with PBS with 0.05% Triton X-100 (PBST), and all immunoreactants were diluted in PBST. For DA determination, DA (17 µg/mL–556 ng/mL, 50 μL) and anti-DA MAbs (10 ng/mL, 50 μL) solutions were poured into the wells. For OA determination, OA (0.9 ng/mL–9 pg/mL, 50 μL) and anti-OA MAbs (50 ng/mL, 50 μL) solutions were poured. The incubation for 1 h at 37 °C was performed for both assays.

Then, the GAMI–HRP conjugate (100 μL, 1:3000 dilution) was poured with the following incubation for 1 h at 37 °C. To measure the HRP activity of the formed complexes, 100 μL of a solution containing TMB (0.42 mM) and H_2_O_2_ (1.8 mM) in 100 mM citrate buffer, pH 4.0, was poured and incubated for 15 min at RT. The reaction was stopped by 1 M H_2_SO_4_ (50 μL per well) and the optical density at 450 nm (OD_450_) was recorded using a Zenyth 3100 vertical photometer (Anthos Labtec Instruments, Wals, Austria).

### 2.5. Synthesis of AuNPs and Choice of GAMI Concentration for Conjugation

AuNPs were obtained by the approach described by Frens (1973) [31] and characterized by transmission electron microscopy (TEM) as reported by Hendrickson et al. (2018) [32] using a CX-100 microscope (Jeol, Tokyo, Japan). The pH of the AuNPs solution (OD_520_ = 1) was adjusted to 9.0. Then, AuNPs (500 μL) and GAMI (0.5–200 μg/mL, 50 μL in 10 mM Tris-HCl, pH 8.5) were mixed with the following incubation for 10 min at RT. Finally, 10% NaCl (50 μL per sample) was added and OD_580_ was measured.

### 2.6. Conjugation of AuNPs with GAMI

For the conjugation, GAMI (6 μg/mL) were added to AuNPs (OD_520_ = 1, pH 9.0). The mixture was shaken for 45 min at RT. After this, 10% BSA was added (1:40, *v*/*v*.) with the additional incubation for 15 min under stirring. Then, the conjugate was pelleted by double centrifugation (9000× *g*, 20 min, 4 °C). The precipitate was resuspended in 10 mM Tris buffer, pH 8.5, with 0.1% NaN_3_, 1% BSA, and 1% sucrose. The finally obtained conjugate (OD_520_ = 15) was stored at 4 °C.

### 2.7. Preparation of Test Strips

A CNPC-SS12 nitrocellulose membrane (Advanced Microdevices, Ambala Cantt, India) was a working membrane, a GFB-R4 membrane from the same manufacturer was a sample pad, and a ReliaFlow 319 membrane (Ahlstrom-Munksjö, Helsinki, Finland) was an adsorption pad.

For individual determination of phycotoxins, DA–BSA or OA–BSA (0.5 mg/mL in PBS) was applied (0.1 μL/mm) to make a test (T) zone. For the double test system, both conjugates were applied in the same concentrations with the formation of two T zones. DAGI (0.1 mg/mL in PBS) were applied to form a control (C) zone. Iso-Flow dispenser from Imagene Technology (Hanover, NH, USA) was used for the application of the regents.

After drying overnight at RT and for 1.5 h at 37 °C, the multimembrane composite was cut using a guillotine (KinBio, Shanghai, China) into strips of 2.9 mm width that were stored in sealed packages with silica gel at RT.

### 2.8. Pretreatment of Seawater and Seafood Samples

Seawater samples were collected from the Aegean Sea (Fethiye region, Turkey) and stored at 4 °C. Before the assays, Triton X-100 (0.05%) was added, and the mixture was 10-fold diluted by PBST and spiked with DA and/or OA.

The tested seafood included octopuses, mussels, tiger shrimps, crabs, whelks, and scallops from local supermarkets. Seafood samples were minced using a blender. To a 0.5 g sample, DA (10 μL, 1 mg/mL, which corresponds to 20 μg/g), OA (160 μL, 1 μg/mL, which corresponds to 320 ng/g), and the methanol–water (1:1) mixture (5 mL) were added. The obtained preparations were stirred for 5 min and centrifuged (1500× *g*, 10 min). The supernatants were 10-fold diluted by PBST and used for the ICA.

### 2.9. Single ICAs of OA and DA

For the determination of DA, its solutions (0.1–2000 ng/mL, 50 μL in PBST), anti-DA MAbs (140 ng/mL, 50 μL in PBST), and GAMI–AuNPs (2 μL, OD_520_ = 15) were mixed. For the determination of OA, its solutions (0.01–66 ng/mL, 50 μL in PBST), anti-OA MAbs (0.1 μg/mL, 50 μL in PBST), and GAMI–AuNPs (2 μL, OD_520_ = 15) were mixed. The mixtures were incubated for 3 min at RT for both cases. Then, the test strips were applied to the mixtures, removed after 15 min, blotted, and scanned using a CanoScan LiDE 90 scanner (Canon, Tokyo, Japan). Bands’ coloration was assessed by TotalLab software from Nonlinear Dynamics (Newcastle upon Tyne, Great Britain).

### 2.10. Double ICA of OA and DA

DA (34 pg/mL–2 μg/mL, 25 μL in PBST), OA (0.4 pg/mL–25 ng/mL, 25 μL in PBST), anti-DA MAbs (304 ng/mL, 25 μL in PBST), anti-OA MAbs (0.2 μg/mL, 25 μL in PBST), and GAMI–AuNPs (5 μL, OD_520_ = 15) were mixed and incubated for 3 min at RT. The test strips were applied to the mixtures for 15 min. All other stages were the same as described above. Spiked seawater or seafood extracts were tested by the same technique.

### 2.11. Evaluation of the ICA and ELISA Results

To build the plots of color intensity or OD (y) versus the analyte concentrations (x) and fit them using a four-parameter logistic function, Origin software from OriginLab (Northampton, MA, USA) was applied. The LODs, working ranges, and cutoffs were determined in the accordance with Hendrickson et al. (2018) and Uhrovcik (2014) [32,33].

## 3. Results and Discussion

### 3.1. Obtaining the Immunoreagents

**Figure 1:**
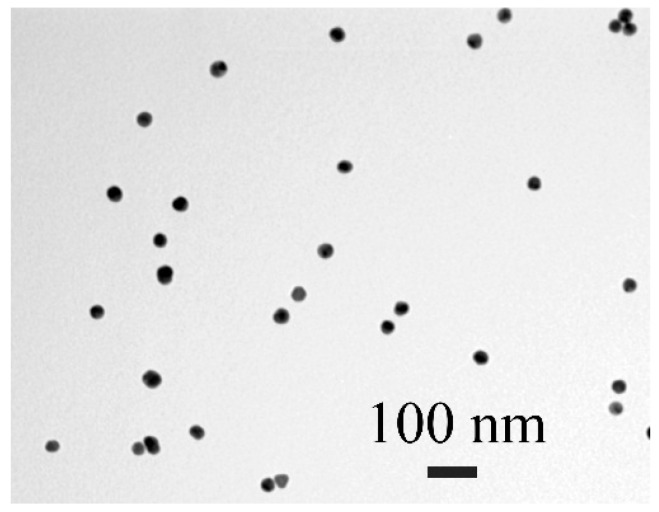
TEM photograph of AuNPs.

AuNPs were used in the developed ICA as a traditional immunochromatographic label characterized by easy synthesis, stability, high sorption capacity, and bright coloration. AuNPs were synthesized by the technique of Frens (1973) based on reducing gold(III) chloride. The reagents ratio, duration of the synthesis, and temperature options ensured the production of nanoparticles with an average diameter near 30 nm, which are considered to be the optimal gold marker in the ICA [34]. The TEM study demonstrated that the AuNPs’ sample contained spherical homogeneous nanoparticles with a diameter of 27.8 ± 3.4 nm and an ellipticity of 1.1 ± 0.2 (Figure 1).

As receptor systems for DA and OA, MAbs with the corresponding specificities were used. Anti-DA antibodies were obtained by immunization of female BALB/c mice using DA conjugates with STI and BSA as immunogens. In the case of OA, commercial antibodies were used. Protein conjugates of OA and DA used as immunogens and coating antigens were obtained by the carbodiimide activation method and purified by dialysis. All obtained conjugates were characterized spectrally. The spectra of DA–STI, DA–BSA, OA–SIT, and OA–BSA had peaks characteristic of haptens and protein components of the corresponding conjugates, which confirmed successful complexation (data not shown). Nine produced clones of MAbs to DA were characterized by the indirect competitive ELISA by titer (defined as the maximal dilution of antibodies that provides reliable binding to the antigen) and the LOD of DA. As a result of testing, the Dom D3 clone was selected, which provides the maximum sensitivity for determining DA (180 ng/mL, Figure S1a). Characterization of the immune interaction of anti-OA MAbs with the synthesized OA–protein conjugates was also carried out by the indirect ELISA. According to the calibration curve (Figure S1b), the LOD of OA in the ELISA was 0.5 ng/mL.

**Figure 2:**
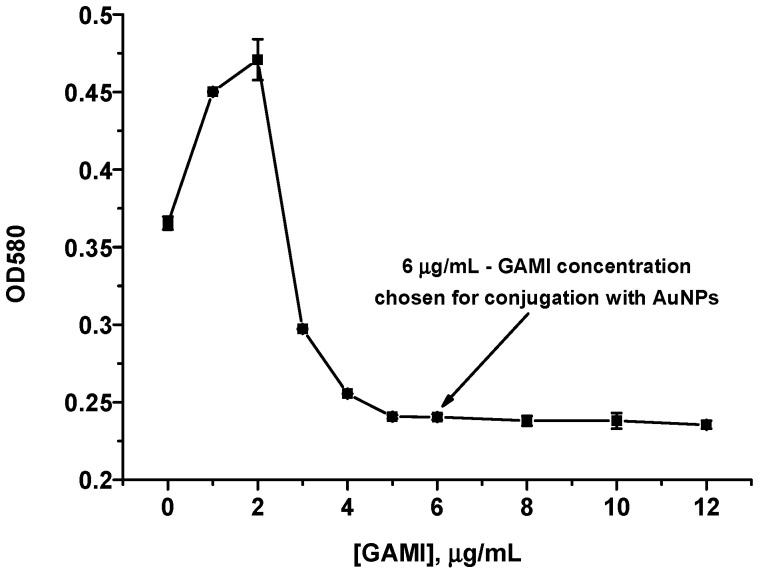
Flocculation curve obtained for GAMI.

Antibodies were labeled with a gold marker by the physical adsorption on the surface of AuNPs. Indirect competitive ICA, which was planned for implementation, involves conjugation with a label not of specific anti-phycotoxin antibodies but anti-species ones (GAMI). To obtain stable GAMI–AuNPs complexes, the added antibodies’ concentration was chosen. For this, a flocculation curve was built reflecting the dependence of AuNPs’ spectral characteristics on the concentration of added immunoglobulins in an environment that promotes the aggregation of non-stabilized AuNPs. At low antibody concentrations, the surface of AuNPs is labile, and the particles aggregate at high ionic strength (10% sodium chloride solution) [35]. Aggregation was followed by a color change of the AuNPs solution to the violet tone and an increase in its OD. Growth of antibodies’ concentration leads to the stabilization of the AuNPs by the adsorbed protein and, accordingly, to a decrease in OD down to a plateau (flocculation point). According to the flocculation curve (Figure 2), the concentration of 6 µg/mL was chosen for the binding of GAMI with AuNPs. The resulting conjugate was stable and retained immunological properties for at least 3 months, providing a high analytical signal in test systems.

### 3.2. ICA of DA

The indirect competitive ICA provides, as a rule, a significant gain in sensitivity and is simply adaptable for multiplex testing due to the lack of need to conjugate each specific antibody [32,36]. To implement the indirect competitive ICA of DA, test strips were obtained based on a multimembrane assembly, which included a nitrocellulose working membrane as the main component. A T and a C zone were made by the application of the DA–protein conjugate and immunoglobulins having specificity to the antibodies in the gold conjugate (namely, DAGI), respectively. The multi-composite test strip did not include a fiberglass conjugate pad with labeled antibodies; therefore, the ICA had two stages. First, specific antibodies and GAMI–AuNPs conjugate were incubated with the sample for several minutes; then the test strip was applied to the reaction mixture. In the absence of DA, the double MAbs–GAMI–AuNPs complex was formed and moved to the T zone and interacted with the immobilized DA–protein conjugate there, forming the first colored line. A surplus of the GAMI–AuNPs conjugate was bound in the C zone to immobilized DAGI, forming a second line. The presence of DA in the sample led to the interaction with specific antibodies (with the formation of a DA–MAbs–GAMI–AuNPs ternary complex), which prevents MAbs from binding to the DA–protein conjugate in the T zone. Therefore, the coloration intensity in the T zone decreases proportionally to the DA concentration increase or is not visualized at all. The excess of the GAMI–AuNPs conjugate, as in the previous case, is concentrated in the C zone.

**Figure 3:**
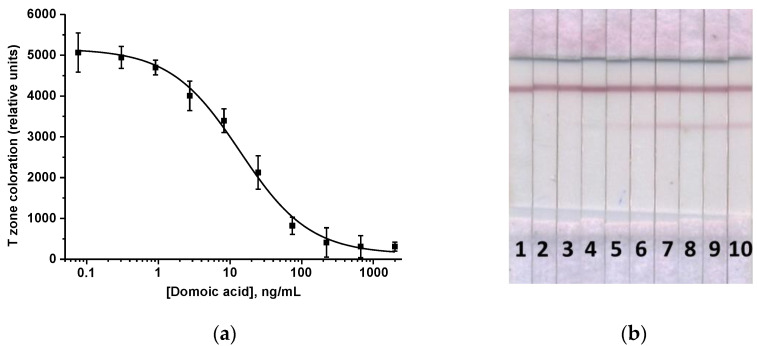
ICA of DA. (**a**) Calibration curve (n = 3). (**b**) The appearance of test strips after the assay. Samples 1–10 accord to 2000, 667, 222, 74, 21, 8.2, 2.7, 0.9, 0.3, and 0.08 ng/mL of DA.

Optimization of the ICA was aimed at maximizing the assay sensitivity and included choosing the concentration of immobilized DA–BSA (the tested range was 0.2–1 mg/mL), DAGI (0.1–0.5 mg/mL), the volume of the added GAMI–AuNPs conjugate (1–6 µL), the duration of the sample incubation with specific and labeled antibodies (the first ICA stage; the tested range was 2–5 min), and the duration of the test strip incubation with the mixed reactants (the second ICA stage; the tested range was 10–20 min). As a consequence, the following ICA options were chosen: DA–BSA and DAGI = 0.5 mg/mL, anti-DA MAb concentration = 140 ng/mL, GAMI–AuNPs volume = 2 μL, the first stage duration = 3 min, and the second stage duration = 15 min. According to the obtained calibration curve, the LOD of DA was 1.2 ng/mL, the visual LOD (cutoff) was 100 ng/mL, and the working range was 2.9–65.1 ng/mL (Figure 3). The assay was completed in 18 min.

### 3.3. ICA of OA

**Figure 4:**
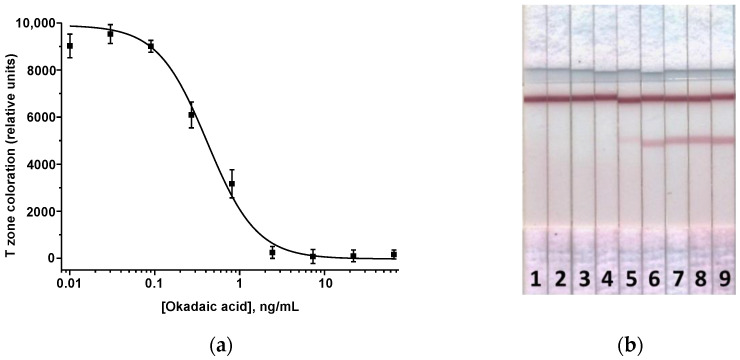
ICA of OA. (**a**) Calibration curve (n = 3). (**b**) The appearance of test strips after the assay. Samples 1–9 accord to 66, 22, 7.3, 2.4, 0.8, 0.3, 0.09, 0.03, and 0.01 ng/mL of OA.

The ICA of OA was implemented in the indirect format with a test system configuration similar to DA, with the same immunoreagents, except for the T zone where OA–protein conjugate was immobilized. The ICA was also optimized to achieve the minimum LOD and high colorimetric signal in test strip zones by varying the assay options as described in Section 3.2. These requirements were met in the following conditions: OA–BSA and DAGI = 0.5 mg/mL, anti-OA MAbs = 0.1 μg/mL, and the GAMI–AuNPs conjugate volume introduced into the sample = 3 μL. Based on the final calibration curve (Figure 4), the LOD was 0.1 ng/mL, the cutoff—2.5 ng/mL, and the working range of detected concentrations—0.16–1.1 ng/mL. The assay was implemented within 18 min.

### 3.4. Double ICA of OA and DA

**Figure 5:**
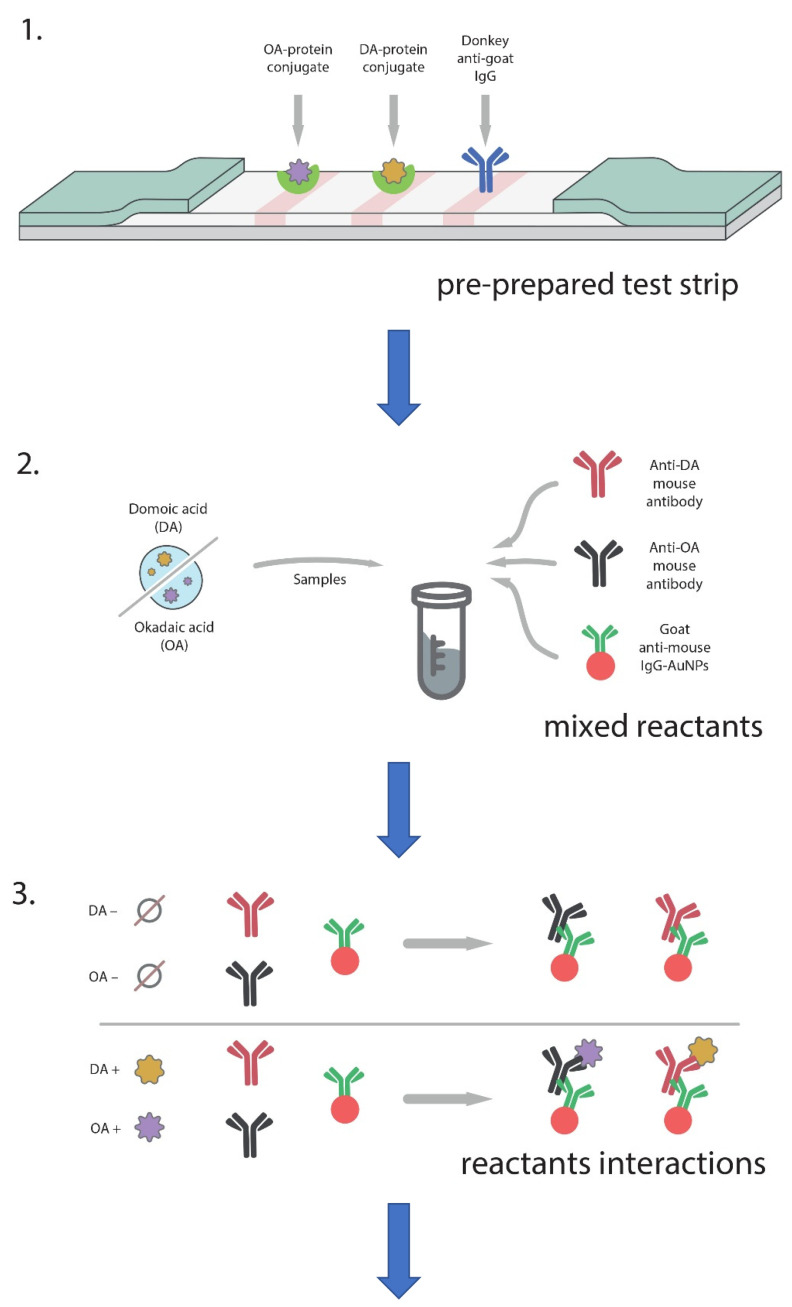

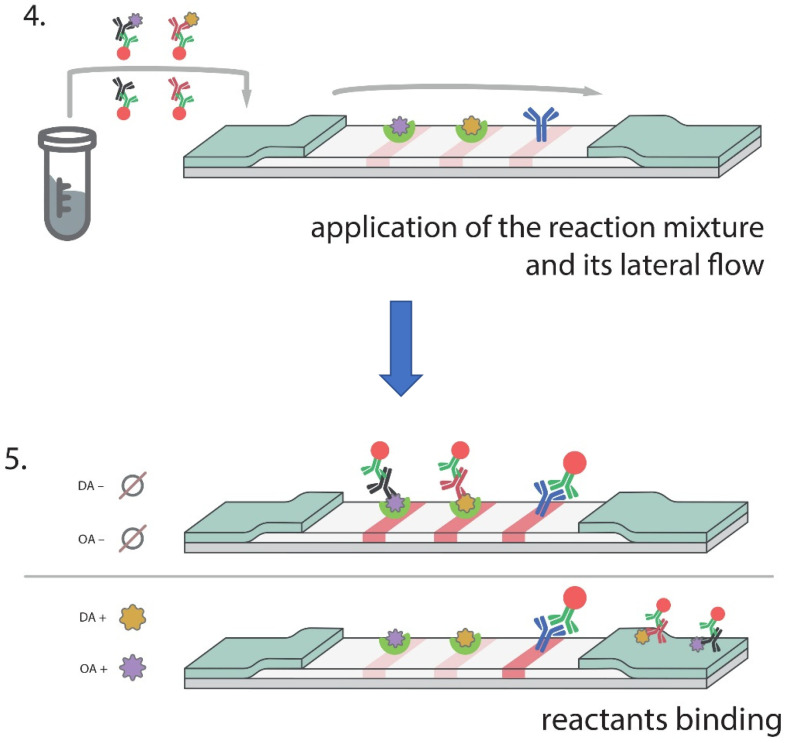
Flow chart for the developed double ICA of DA and OA. 1: test strips for the double ICA of DA and OA are obtained; 2: reaction mixtures containing a tested sample, anti-DA, anti-OA specific MAbs, and GAMI–AuNPs are prepared; 3: immune interactions occur in the reaction mixtures; 4: test strips are immersed in the reaction mixtures; 5: liquid flow moves along the test strip followed by the immune interactions on the working membrane.

The development of a multiplex test system was carried out, taking into account the results obtained when creating individual tests for DA and OA. To implement a multiparametric immunochromatographic test system, it is necessary to form several T zones on a working membrane following the number of analyzed compounds. Thus, for the simultaneous detection of DA and OA, three zones were formed: two T zones with immobilized OA–BSA and DA–BSA conjugates and a C zone with anti-species antibodies (the flow chart is shown in Figure 5). Because both anti-DA and anti-OA antibodies are monoclonal, one type of anti-species antibody was applied in the C zone (DAGI). When conducting a multi-analysis, all components of the test system were added to the reaction mixture simultaneously, but in smaller amounts compared to single tests, to maintain the total volume of the reaction medium unchanged. Namely, at the first stage of the ICA, two analyzed samples, two types of specific antibodies, and a GAMI–AuNPs conjugate were mixed and preincubated; then, test strips were placed in the mixture.

**Figure 6:**
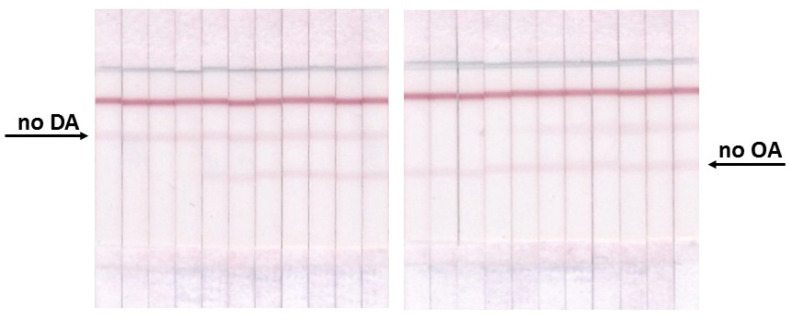
Images of test strips for double ICA in the absence of one of the analytes.

Taking into account the possible mutual influence of immunoreagents and changes in their volumes and concentrations in the reaction mixture compared to individual tests, additional steps are required to select the optimal conditions for the implementation of multi-analysis. Such a choice was started with the study of the influence of the location of T zones on the analytical parameters of the system. The following preliminary arrangement of zones was used: T1 zone (OA–BSA) → T2 zone (DA–BSA) → C zone. With this arrangement of zones, the concentrations of immobilized reagents used for individual tests were used. Then, the ICA was carried out under the same conditions with a different arrangement of zones: T1 zone (DA–BSA) → T2 zone (OA–BSA) → C zone. According to the data obtained, the LOD of phycotoxins and signal intensities did not reliably differ in both variants of T zone arrangement; therefore, for subsequent experiments, test systems with the T1 zone (OA–BSA) → T2 zone (DA–BSA) → C zone arrangement were used. Control experiments were conducted, in which double testing was carried out in the absence of one of the analytes. When there was no phycotoxin in the sample, the uniform coloration in the corresponding T zone was observed (Figure 6).

**Figure 7:**
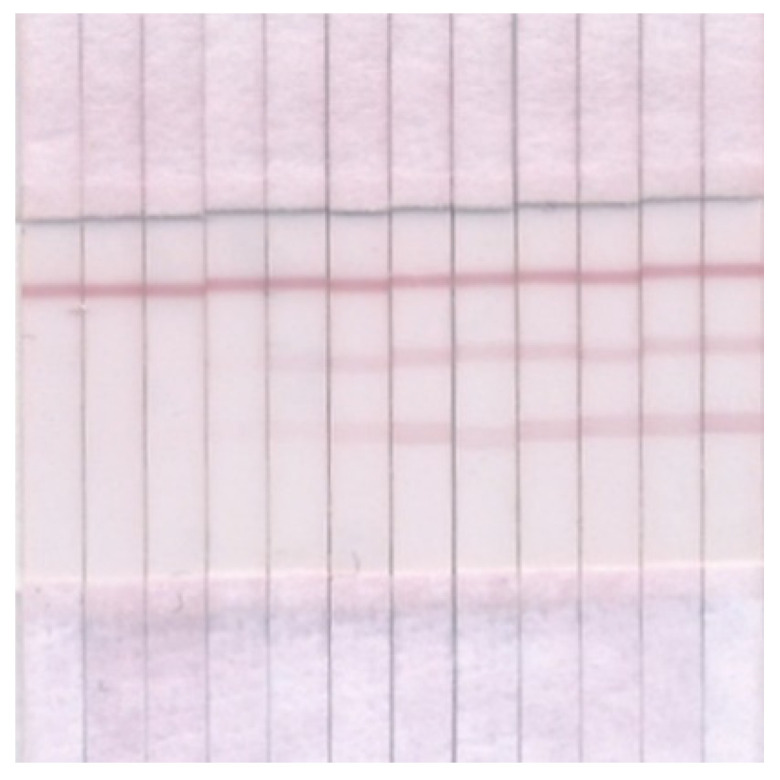
Images of test strips for double ICA of DA and OA. DA/OA concentrations from left to right: 2000/25, 667/8.3, 222/2.8, 74/0.9, 25/0.3, 8.2/0.1, 2.7/0.03, 0.91/0.01, 0.3/3.8 × 10^−3^, 0.1/1.3 × 10^−3^, 0.03/4.2 × 10^−4^, and 0/0 ng/mL.

The optimization of the test system showed that some of the conditions coincided with those for the individual determination of each phycotoxin (namely, concentrations of immobilized DA–BSA and OA–BSA), and some had to be changed to achieve the maximum sensitivity of the analysis, taking into account changes in the lineament of the test system. Thus, the volume of the label was increased to 5 µL, because in the double test there are three label-binding zones, and with an insufficient amount of the label, the coloration in the T zones is not intense enough to ensure the accuracy and reproducibility of the analysis. However, with this amount of GAMI–AuNPs, the band coloration in the C zone is disproportionately higher than in the T zones (Figure 6), so the DAGI concentration was reduced to 0.1 mg/mL. The amount of antibodies was increased approximately 2 times compared to individual tests (0.2 μg/mL for anti-OA MAbs and 304 ng/mL for anti-DA MAbs) because the quantity of added antibodies was reduced by half in a double test. The time of preincubation of the reagents before the introduction of the test strips and the time of the test strips’ incubation with the mixed reactants did not change, and it amounted to 3 and 15 min, respectively. Under conditions optimized in this way, LODs in the double test and in the individual tests did not differ (see Figure 7).

### 3.5. ICA of OA and DA in Seawater

For the double immunochromatographic detection of DA and OA, a sample of natural seawater was taken from the Aegean Sea. A series of dilutions of DA and OA were made in untreated seawater, and the ICA was performed as described in Section 2.10. It was shown that a large amount of GAMI–AuNPs was retained on the sample pad and only partially moved with the liquid flow (Figure S2). Therefore, seawater was diluted 2 and 10 times with PBST; then, dilutions of DA and OA were prepared in the resulting solutions and analyzed. With this seawater pretreatment, the matrix influence was suppressed, and the retention of labeled conjugate at the lower edge of the test strip decreased with increasing water dilution with PBST (Figure S2). However, in comparison with the ICA in the model system (buffer), the LODs and the amplitude of the colorimetric signal changed significantly. Moreover, these dependencies were different for DA and OA detection.

Permanent background coloration was observed in the T zone with immobilized DA–BSA and signal intensity was practically independent of the DA concentration, both at the 2-fold and 10-fold dilutions of seawater with PBST (Figure S2). Generally, the amplitude of the colorimetric signal increased by ~60–80%, compared to the signal amplitude upon detection in the buffer regardless of dilution (Table S1). Therefore, it was not possible to assess the DA LOD in undiluted and PBST-diluted seawater due to the high background (Figure S2, Table S1). In the case of OA detection, signal intensity decreased by 30% for undiluted water and by 20% for water diluted 2 times with PBST. At a 10-fold dilution, the amplitude value corresponding to that detected in the buffer. Under these conditions, an LOD and a cutoff of OA correlated with those measured in the model conditions. Therefore, for the reliable detection of OA, a simple dilution of natural seawater by 10 times with PBST is enough. However, such conditions are not suitable for DA detection, and as far as the test system is designed for the simultaneous control of two phycotoxins, we implemented a further selection of conditions that meet the requirement for efficient seawater processing before analysis.

Because the mobility of reagents and nonspecific effects in immunochromatography directly depends on the composition of the medium, particularly in the presence of a detergent, Triton X-100 (0.05%) was added to the seawater. The samples were diluted 2, 5, and 10 times with PBST, spiked with DA and OA, and analyzed by double ICA. As a result, it was shown that the signal amplitude in the T zone with immobilized DA–BSA was the same when detected in PBST and in water samples diluted 2, 5, or 10 times (Table S2). In the T zone with immobilized OA–BSA, a correspondence is observed only when detected in PBST and seawater with the maximum dilution. LODs of OA and DA did not differ significantly, regardless of sample preparation. The cutoff of DA increased by several times when the water was diluted by 2 and 5 times, i.e., the sensitivity of the analysis deteriorated. Considering all the results obtained, the optimal conditions for seawater sample preparation for simultaneous detection of DA and OA is the preliminary addition of 0.05% Triton X-100 detergent, followed by a 10-fold dilution of water with PBST (rows highlighted in gray in Table S2).

For each zone, the selectivity of the label binding was shown. When testing samples containing three widespread phycotoxins, OA, DA, or MC-LR, only the target analyte, unlike the other two compounds, resulted in a statistically significant decrease in coloration intensity. In addition, the course of the concentration dependence for OA did not change if DA was simultaneously present in the sample in different concentrations, and vice versa, DA did not influence the coloration of the OA-specific binding zone.

Under the selected conditions of sample preparation, the recoveries of DA and OA in seawater were determined. First, Triton X-100 was added to the samples. Then, the samples were spiked with DA and OA and 10-fold diluted by PBST to obtain final concentrations within the working ranges (0.2 ng/g, 0.4 ng/g for OA and 8 ng/g, 20 ng/g for DA), and testing was carried out. The recovery values of 109.4–121.5%. (see Table 1) indicate that the test system is suitable for the determination of DA and OA in seawater.

### 3.6. ICA of OA and DA in Seafood

A large panel of seafood included both commonly eaten seafood, such as tiger prawns, mussels, and octopuses, and delicacies, such as whelks, crabs, and scallops. The absence of DA and OA in seafood was confirmed by ELISA kits (EuroProxima, Arnhem, The Netherlands). Because seafood cannot be analyzed directly, sample preparation was required before analysis. This is aimed at reducing the matrix effect and enhancing the efficient extraction of phycotoxins. The literature analysis demonstrated that both DA and OA could be effectively extracted by a methanol–water mixture without any additional treatment (Hu et al., 2013; Liu et al., 2014; Lu et al., 2012). For sample preparation, homogenized seafood was spiked with DA and/or OA. A water–methanol mixture (extractant) was added, mixed, and centrifuged, and the supernatant was isolated. Such sample preparation took only 20 min. A clear obtained extract was 10-fold diluted with PBST and used for the analysis. Recovery coefficients were determined for three concentrations for each phycotoxin. The obtained values are presented in Table 2.

As can be seen from the presented data, the test system enables the determination of 80.8–124.1% phycotoxins. The accuracy of the assay was confirmed using the ELISA kits from EuroProxima. High coefficients of correlation (0.981, n = 10) between the amounts of phycotoxins determined by the ICA and the ELISA for seawater and seafood extracts were reached. The advantages of this test system over published ones include a new combination of relevant phycotoxins for simultaneous detection, high sensitivity (beyond official requirements), and a very short sample preparation procedure, which allows for the rapid screening of real samples. In addition, unlike previous works, the analysis of real objects was not limited to one or two types of seafood but was carried out with a large panel of samples. The listed above features confirm the competitive potential of the proposed test system.

## 4. Conclusions

The study demonstrates the proof-of-principle for the immunoanalytical test system providing simultaneous detection of DA and OA as two dangerous marine toxins. The test system is based on the indirect competitive ICA with gold-labeled secondary antibodies. The LODs/cutoffs/linear ranges of the ICA were 1.2/100/2.9–65.1 and 0.1/2.5/0.16–1.1 ng/mL for DA and OA, respectively, with a testing duration of 18 min. The proposed method of sample preparation for seafood was very simple and allowed for the processing of a complex matrix within 20 min. For seawater, only a simple dilution with a buffer was required. Thus, the total assay time (from obtaining a sample to assessing the ICA result) is only 40 min or less (when analyzing seawater), which confirms the promise of the assay for rapid and easy determination of phycotoxins in various objects. The relevance and importance of the presented research are caused by its focus on two different kinds of marine biotoxins (belonging to ASP and DSP groups) detected with a single test. For future tasks, the applicability of the developed test on real biotoxin-containing food and water samples can be considered.

## Figures and Tables

**Table 1:** Recoveries of DA and OA from seawater (n = 3).

Phycotoxin	Added Quantity (ng/g)	Measured Quantity ± SD (ng/g)	Recovery ± ^1^ SD (%)
DA	20	23.6 ± 0.96	118.2 ± 4.8
	8	8.75 ± 0.35	109.4 ± 4.4
OA	0.4	0.49 ± 0.01	121.5 ± 2.9
	0.2	0.24 ± 0.009	118.1 ± 4.6

^1^ SD = standard deviation.

**Table 2:** Recoveries of DA and OA from seafood (n = 3).

Domoic Acid			
Matrix	Added Quantity (µg/g)	Measured Quantity ± SD (µg/g)	Recovery ± ^1^ SD (%)
Scallops	20	17.4 ± 1.3	87.4 ± 6.4
	8	6.7 ± 0.7	83.4 ± 9.2
	3.2	2.8 ± 0.2	88.8 ± 7.3
Tiger shrimps	20	23.2 ± 1.6	116.2 ± 7.8
	8	9.2 ± 0.4	114.7 ± 5.3
	3.2	2.8 ± 0.3	115.1 ± 8.8
Whelks	20	18.2 ± 1.2	91.0 ± 5.8
	8	7.1 ± 1.1	88.8 ± 13.9
	3.2	2.6 ± 0.2	80.8 ± 5.0
Octopuses	20	20.6 ± 0.9	102.9 ± 4.5
	8	9.9 ± 0.2	123.3 ± 2.7
	3.2	3.7 ± 0.3	116.4 ± 8.2
Mussels	20	22.1 ± 0.3	110.6 ± 1.7
	8	9.6 ± 0.3	119.8 ± 3.2
	3.2	4.0 ± 0.3	124.1 ± 7.8
Mussels	20	22.9 ± 3.0	114.5 ± 15.0
	8	8.9 ± 1.2	111.9 ± 14.7
	3.2	3.8 ± 0.1	120.0 ± 3.1
**Okadaic acid**			
	Added quantity (ng/g)	Measured quantity ± SD (ng/g)	Recovery ± ^1^ SD (%)
Scallops	320	265.3 ± 32.0	82.9 ± 10
	160	130.7 ± 6.2	81.7 ± 3.9
	80	71.9 ± 5.2	89.9 ± 6.5
Tiger shrimps	320	295.7 ± 46.4	92.4 ± 14.5
	160	165.3 ± 9.1	103.3 ± 5.7
	80	89.3 ± 1.9	111.6 ± 2.4
Whelks	320	291.5 ± 25.6	91.1 ± 8.0
	160	171.8 ± 9.3	107.4 ± 5.8
	80	66.2 ± 6.1	82.7 ± 7.6
Octopuses	320	376.6 ± 48	117.7 ± 15.0
	160	190.2 ± 22.9	118.9 ± 14.3
	80	88.4 ± 7.4	110.5 ± 9.3
Mussels	320	389.4 ± 18.2	121.7 ± 5.7
	160	184.3 ± 2.4	115.2 ± 1.5
	80	97.4 ± 3.7	121.8 ± 4.6
Crabs	320	394.9 ± 13.4	123.4 ± 4.2
	160	162.4 ± 18.9	101.5 ± 11.8
	80	84.2 ± 10.1	105.2 ± 12.6

^1^ SD = standard deviation.

## Data Availability

Data are contained within the article. Initial data of instrumental measurements are available on request from the corresponding author.

## Supplementary Materials

The following supporting information can be downloaded at: https://www.mdpi.com/article/10.3390/mi13091506/s1, Figure S1: Calibration curves of DA (a) and OA (b) in the indirect ELISA; Figure S2: Images of the test strips after the double ICA of DA and OA in seawater; Table S1: Comparison of analytical characteristics of double ICA for different methods of seawater sample preparation.; Table S2: Comparison of the analytical characteristics of double ICA with different methods of sample preparation of seawater containing Triton X-100.
