# Supplementary material for: Double Immunochromatographic Test System for Sensitive Detection of Phycotoxins Domoic Acid and Okadaic Acid in Seawater and Seafood
Source: Micromachines (Basel). 2022 Sep 10;13(9):1506. doi: 10.3390/mi13091506 (PMC9505318; doi:10.3390/mi13091506)
Supplement: Supplementary file 1 [file micromachines-13-01506-s001.zip › micromachines-1886622-supplementary.pdf]

*micromachines*

*Article*

# Double Immunochromatographic Test System for Sensitive Detection of Phycotoxins Domoic Acid and Okadaic Acid in Seawater and Seafood

**Olga D. Hendrickson [1], Elena A. Zvereva [1], Olga N. Solopova [2], Anatoly V. Zherdev [1], Peter G. Sveshnikov [3], Sergei A. Eremin [1,4,*], and Boris B. Dzantiev [1]**

[1] Bach Institute of Biochemistry, Research Center of Biotechnology of the Russian Academy of Sciences, Leninsky Prospect 33, 119071 Moscow, Russia
[2] Blokhin National Medical Research Center of Oncology, Ministry of Health of the Russian Federation, Kashirskoye Shosse 24, 115478 Moscow, Russia
[3] Russian Research Center for Molecular Diagnostics and Therapy, Sympheropolsky Blvrd., 8, 117638 Moscow, Russia
[4] Faculty of Chemistry, Lomonosov Moscow State University, Leninskie Gory, 119991 Moscow, Russia
* Correspondence: saeremin@gmail.com; Tel.: +79165127654

**Citation:** Hendrickson, O.D.; Zvereva, E.A.; Solopova, O.N.; Zherdev, A.V.; Sveshnikov, P.G.; Eremin, S.A.; Dzantiev, B.B. Double Immunochromatographic Test System for Sensitive Detection of Phycotoxins Domoic Acid and Okadaic Acid in Seawater and Seafood. *Micromachines* **2022**, *13*, 1506. https://doi.org/10.3390/mi13091506

Academic Editor: Elizaveta Panfilova

Received: 11 August 2022
Accepted: 9 September 2022
Published: 10 September 2022

**Publisher's Note:** MDPI stays neutral with regard to jurisdictional claims in published maps and institutional affiliations.

**Copyright:** © 2022 by the authors. Licensee MDPI, Basel, Switzerland. This article is an open access article distributed under the terms and conditions of the Creative Commons Attribution (CC BY) license (https://creativecommons.org/licenses/by/4.0/).

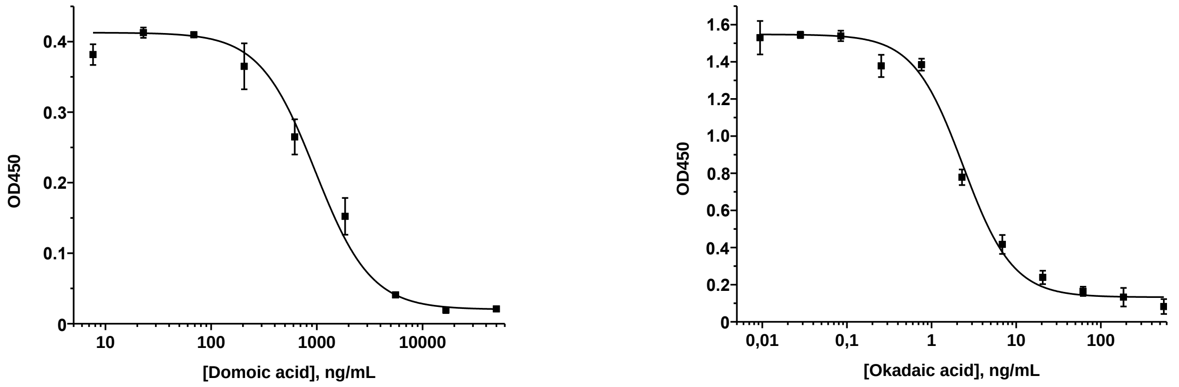


(**a**) (**b**)

**Figure S1.** Calibration curves of DA (**a**) and OA (**b**) in the indirect ELISA.

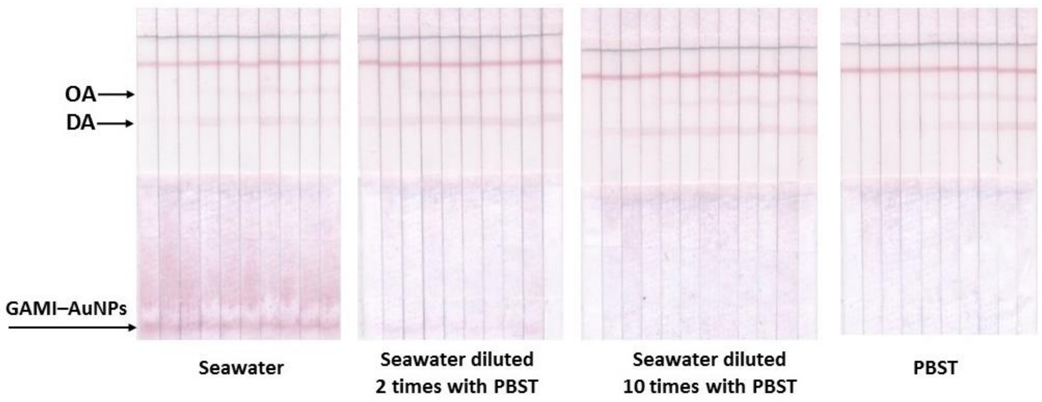


**Figure S2.** Images of the test strips after the double ICA of DA and OA in seawater.

**Table S1.** Comparison of analytical characteristics of double ICA for different methods of seawater sample preparation.

| ICA conditions | Maximum signal amplitude, % | LOD, ng/mL | Cutoff, ng/mL |
|---|---|---|---|
| ICA of DA/OA in PBST | 100/100 | 1.2/0.1 | 100/2.5 |
| ICA of DA/OA in undiluted seawater | 177/70 | n.d./0.1 | n.d./2.5 |
| ICA of DA/OA in seawater diluted 2 times with PBST | 156/80 | n.d./0.1 | n.d./2.5 |
| ICA of DA/OA in seawater diluted 10 times with PBST | 156/100 | n.d./0.1 | n.d./2.5 |

**Table S2.** Comparison of the analytical characteristics of double ICA with different methods of sample preparation of seawater containing Triton X-100.

| ICA conditions | Maximum signal amplitude, % | LOD, ng/mL | Cutoff, ng/mL |
|---|---|---|---|
| ICA of DA/OA in PBST | 100/100 | 1.4/0.1 | 100/2.5 |
| ICA of DA/OA in seawater diluted 2 times with PBST | 100/150 | 1.4/0.1 | 400/2.5 |
| ICA of DA/OA in seawater diluted 5 times with PBST | 100/150 | 1.4/0.1 | 400/2.5 |
| ICA of DA/OA in PBST | 100/100 | 1.4/0.1 | 100/2.5 |
